# Prevalence and risk factors of postpartum depression among women attending primary healthcare centers in northern of West Bank/ Palestine: a cross-sectional study, 2022

**DOI:** 10.1186/s12905-024-02887-6

**Published:** 2024-01-15

**Authors:** Dina Wildali, Saja Nazzal, Suha Hamshari, Souad Belkebir

**Affiliations:** 1 Department of Family and Community Medicine, Faculty of Medicine and Health Sciences, An-Najah National University, Nablus, Palestine; 2 Department of Family and Community Medicine, Faculty of Medicine and Health Sciences, An-Najah National University, Nablus, Palestine; 3 Department of Family and Community Medicine, Faculty of Medicine and Health Sciences, An-Najah National University, Nablus, Palestine; 4 Department of Family and Community Medicine, Faculty of Medicine and Health Sciences, An-Najah National University, Nablus, Palestine

**Keywords:** Edinburgh postnatal depression scale, Postpartum depression, Primary healthcare, Risk factors, West Bank

## Abstract

**Background:**

Postpartum depression (PPD) has a huge negative impact on the health of the mother and the family, both physically and mentally. Few postpartum depression studies have been done in Palestine. This study aimed to examine the prevalence and the most probable risk factor of PDD among Palestinian women in the northern West Bank.

**Methods:**

This is a cross-sectional study of 380 mothers, ages 18 and 44 years, visiting vaccination clinics with their infants after 7-12 weeks of delivery between 1 May 2022 and 30 June 2022. Postpartum women seeking care at the seven largest primary health care centers of the Ministry of Health in four cities in the Northern West Bank in Palestine were asked to complete a self-administered questionnaire that included the Edinburgh Postnatal Depression Scale and demographic and birth details. A score of 13 or higher was used to indicate PPD risk. Descriptive and analytical analyses were performed using SPSS version 20. The level of significance was set at 5%.

**Results:**

The median age of the participants was 27 years with a range of 26 years. A total of 129 women had an EPDS score of 13 or more, giving a prevalence rate of post-partum depression of 33.9%. The predictors of postpartum depression were stressful life events during pregnancy (*p*-value 0.003, OR: 2.1, 95% CI [1.27-3.4]), vacuum use during delivery *p*-values 0.002, OR: 4, 95% CI: [1.64-9.91]), low social support (*p*-value less than 0.001, OR: 2.5, 95%CI: [1.7-4.2]) and husband’s low level of education (*p*-value less than 0.001, OR: 5.2, 95%CI: [2.7-10]).

**Conclusion:**

The study showed a high prevalence of PPD among Palestinian mothers in the northern West Bank. Our study found that PPD risk factors include lack of social support, the husband’s low education, and stressful events during pregnancy. This will emphasize the importance of PPD screening and early intervention, especially among vulnerable women.

## Introduction

Women go through emotional and psychological changes during the perinatal period making them more susceptible to psychiatric disorders, such as postpartum depression [1].

Postpartum depression may impair parenting skills and judgment [2], decrease enjoyment of the maternal role, and create poor mother-infant interactions. Mothers with PPD show an early cessation of breastfeeding and less care of their infants which leads to decreased immunity and puts babies at risk for delayed growth and development [3]. Moreover, maternal mental illness can also affect a child’s emotional and cognitive development [4]. Undetected PPD imposes a heavy cost on society since the mother is less able to fulfill her responsibility as a caregiver [5].

The Diagnostic and Statistical Manual of Mental Disorders defines postpartum depression as the “Occurrence of a major depressive episode (MDE) within four weeks after birth, which may involve irritability, excessive crying, or panic” [6]. However, these episodes might begin or persist during the first year after delivery [7].

In low- and middle-income countries, PPD affects up to 48.5% of women [4], but just 6.5–12.9% of women in high-income nations [7]. Postpartum depression has been linked to several risk factors, including stressful life events, a history of depression, not breastfeeding, the first delivery, and having a poor body image. Other risk factors include having a poor relationship with their partner and having a lower socioeconomic status [8]. in some contexts, however, higher education, permanent employment, a kind, trustworthy partner, and belonging to the majority ethnic group were protective factors against PPD [1].

In the West Bank, a cross-sectional study conducted in Nablus city in 2013 showed a prevalence of 17% with depression during pregnancy and a history of mental illness being the highest associated risk factors [9]. Another study in Bethlehem in 2016 showed a prevalence of 27.7%, with multiparty and unplanned pregnancy having the highest association with PPD [8].

A recent systematic analysis of the frequency of postpartum depression in Arab nations revealed that 1 in 5 women experience the condition, with some of the most common risk factors being low socioeconomic status, unwanted pregnancy, low social and husband support, stressful life events during pregnancy and personal or family history of depression [10].

A variety of instruments have been used to screen for PPD including the Beck Depression Inventory (BDI) and the Mini International Neuropsychiatric Interview (MINI) [11]. However, EPDS is most suited for postnatal and primary care settings [12] and several studies have been done to assess its validity in varying cut-off scores [13–15]. The USPTFS recommends screening pregnant and post-partum women with EPDS but doesn’t specify a cut-off value [16].

Despite the impact of post-partum depression, few studies have been conducted in Palestine.

### Objectives

This study aims to investigate the prevalence of postpartum depression among Palestinian women attending vaccination clinics at primary health care centers in the northern of west bank in 2022 and to pinpoint risk factors for the condition.

## Methods

### Study design and setting

This study is a descriptive cross-sectional study that was conducted in the northern West Bank between 1 May 2022 and 30 June 2022.Women visiting vaccination clinics at the seven largest primary health care centers of the Ministry of Health in four cities (Nablus, Tulkarem, Jenin, and Qalqelia) with their infants after 7-12 weeks of delivery were asked to participate.

These clinics are the only to provide vaccination services in the four cities (2 in Nablus, 2 in Tulkarem, 2 in Jenin, 1 in Qalqelia,) and post-partum women are expected to be seen there with their infants. Mother who agreed to participate were consented and given a self-administered questionnaire including the Arabic version of EPDS This instrument is commonly employed as a self-administered questionnaire for screening postpartum depression [2]. Additionally, studies have demonstrated comparable efficacy between self-administered questionnaires and face-to-face interviews for diagnosing this particular condition [3].

To ensure the data quality, one of the researchers was present at the site of data collection to ensure that the questionnaire was comprehensive. To ascertain the integrity of the data, the researcher examined the questionnaires completed by the participants for both completeness and consistency. Incomplete questionnaires were discarded.

### Inclusion criteria

Women who were between 18 and 44 years old, 7-12 weeks after delivery, and able to read the Arabic version of the questionnaire were recruited to participate.

### Exclusion criteria

Women who experienced depression or used antidepressants medication.

### Sample size and sampling method

The sample size was calculated with the Raosoft sample size calculator. Based on the annual report of the Palestinian Central Bureau of Statistics, there were 28,273 live births in the northern of West Bank in 2020 [17]. The minimum sample size was 380 at 95% confidence level, 5% margin of error and 50% response distribution.

In order to have a representative sample of the target population to achieve the aim of the study, a proportionate sample was calculated as shown in Table 1. Then, a convenient sample was collected from women presenting to the vaccination clinics at the largest primary care centers in West Bank/Palestine in May and June, 2022. This study was done in primary care. The facility has a psychological department. This department is aimed for persons with mental health concerns who require extra help. Before starting our research, we communicated with the psychiatric department and agreed to manage patients with various depression levels. All positive screening results were forwarded for further testing.

**Table 1:** Association between PPD and Socio-demographic characteristics. (*n* = 380)

Variable	Categories	EPDS ≥ 13	EPDS < 13	Total	^**+**^***P*** value
**Age(years)**	**18-25**	47 (36.4%)	93 (37.1%)	140 (36.8%)	0.704
	**26-35**	69 (53.5%)	139 (55.4%)	208 (54.7%)	
	**36-44**	13 (10.1%)	19 (7.5%)	32 (8.4%)	
	**Median:27**				
**Age at marriage(years)**	≤ **25**	102 (79.1%)	218 (86.8%)	320 (84.2%)	0.109
	**26-35**	24 (18.6%)	31 (12.4%)	55 (14.5%)	
	**≥ 36**	3 (2.3%)	2 (0.8%)	5 (1.3%)	
	**Median:22,**				
**Residence**	**Refugee Camp**	2 (1.6%)	10 (4.0%)	12 (3.1%)	0.362
	**Village**	15 (11.6%)	34 (13.5%)	49 (12.9%)	
	**City**	112 (86.8%)	207 (82.5%)	319 (84%)	
**Education (mother)**	**≤ 6**	3 (2.3%)	16 (6.4%)	19 (5%)	0.166
	**7-12**	36 (27.9%)	58 (23.1%)	94 (24.8%)	
	**> 12 years**	90 (69.8%)	177 (70.5%)	267 (70.3%)	
**Occupation**	**Housewife**	97 (75.2%)	205 (81.7%)	302 (79.5%)	0.334
	**Employed**	27 (21.0%)	39 (15.5%)	66 (17.4%)	
	**other**	5 (3.8%)	7 (2.8%)	12 (3.2%)	
**Insurance**	**Non**	46 (35.7%)	72 (28.7%)	118 (31.1%)	0.481
	**Governmental**	58 (44.9%)	118 (47.0%)	176 (46.3%)	
	**Private**	14 (10.9%)	37 (14.7%)	51 (13.4%)	
	^a^**NGOs**	11 (8.5%)	24 (9.6%)	35 (9.2%)	
**Income(dollar)**	**< 400,400**	7 (5.4%)	18 (7.2%)	25 (6.6%)	0.804
	**400 – < 800**	72 (55.8%)	139 (55.4%)	211 (55.5%)	
	**≥ 800**	50 (38.8%)	94 (37.4%)	144 (37.9)	
	**Median:3000**				
**Education (husband)**	**≤ 6**	39 (30.2%)	30 (12.0%)	69 (18.2%)	< 0.001
	**7-12**	45 (34.9%)	85 (33.8%)	130 (34.2%)	
	**> 12 years**	45 (34.9%)	136 (54.2%)	181 (47.6%)	
**Actual marital status**	**Still married**	129 (100.0%)	249 (99.2%)	378 (99.4%)	0.597
	**Separated**	0 (0.0%)	1 (0.4%)	1 (0.3%)	
	**Other**	0 (0.0%)	1 (0.4%)	1 (0.3%)	

^+^ Chi-squared test; Significant level at *p* < 0.05. ^a^Nongovernmental organization

### Instrument

Data was collected using a 3 part self-administered questionnaire; 1) A pre-defined checklist used to collect data about socio-demographic factors, pregnancy and birth-related factors, baby-related factors, and psychological history [9]. 2) The Maternal Social Support Scale (MSSS), which is a 6-question 5 point-Likert scale used to assess the social support mothers were given after giving birth. The possible highest score is 30 with a score of 6-18 on the MSSS considered low social support, 19-24 medium support, and > 24 adequate support. The reliability of the scale (Cronbach’s alpha) was 0.71-0.90 [18]. 3) The Arabic version of the Edinburgh Postnatal Depression Scale (EPDS), a self-reporting screening tool, composed of 10 items reflect the mother’s emotional experience over the past 7 days. Responses were scored 0–3 indicating the severity of manifestations, with a maximum score of 30.

There is a debate on the single cut-off with the highest sensitivity and specificity. No studies were conducted in Palestine to examine the EPDS validity and reliability. However, a recent systematic review in the Arab world showed that most studies used 13 or greater as a cut off to maximize consistency with other studies. The validity and reliability of the Arabic version of the Edinburgh Postnatal Depression Scale were reviewed with internal reliability of .84 (alpha Cronbach) [12].

### Data analysis

Data were entered into Excel and analyzed using SPSS program version 20. We used univariate descriptive analysis for all variables.

Bivariate analysis was used to study the relationship between dependent and independent variables and test the null hypothesis. *P* value< 0.05 was considered significant.

All significant variables were exported to multivariate analysis using logistic regression to attenuate the effect of cofounders.

### Ethical consideration

All methods involving human participants in this study were conducted per ethical research standards. The study was conducted in conformity with the ethical norms of An-Najah National University (ANNU). The Ministry of Health approved authorization for the study to be conducted in PHC settings, and participants were approached and invited voluntarily to participate. Participants were assured of their confidentiality and anonymity. The ethical code Med. March 8/2022.

## Results

### Descriptive results

#### Characteristics of participants

From the 420 questionnaires that were distributed, 390 patients responded. Ten were excluded due to insufficient data. A total of 380 women with the median age of 27 and a range of 26, with the majority of them falling between the ages of 26 and 35 (*n* = 208, 54.7%) participated.

The majority (84%, *n* = 319) were city dwellers, highly educated (studied beyond secondary school) (70.3%, *n* = 267), and married to highly educated men (47.6%, *n* = 181).

Additionally, most of them were still married at the time of the research (99.5%, *n* = 378), had government insurance (46.3%, *n* = 176), and were unemployed (79.5%, *n* = 302). Their salary ranged from (400 $) to less than (800$) (55.5%, *n* = 211) middle income. See Table 1.

### Birth and child related characteristics of the participants

As Tables 2 and 3 show, most of the participants were multiparas (68.7%, *n* = 261), without any previously diagnosed chronic illness (87.1%, *n* = 331).

**Table 2:** Association between Birth-related Factors and PPD

Variable	Categories	EPDS ≥ 13	EPDS < 13	Total	^**+**^***P*** value
**Prim parity**	**Yes**	53 (41.1%)	66 (26.3%)	119 (31.3%)	0.003
	**No**	76 (58.9%)	185 (73.7%)	261 (68.7%)	
**Complications**	**Yes**	55 (42.6%)	86 (34.3%)	141 (37%)	0.110
	**No**	74 (57.4%)	165 (65.7%)	239 (63%)	
**Place of birth**	**Governmental**	41 (31.8%)	87 (34.7%)	128 (33.7%)	0.472
	**Private**	84 (65.1%)	149 (59.4%)	233 (61.3%)	
	**Agency**	4 (3.1%)	13 (5.2%)	17 (4.5%)	
	**Others**	0 (0.0%)	2 (0.7%)	2 (0.5%)	
**Type of birth**	**Vaginal**	63 (48.8%)	123 (49.0%)	186 (48.9%)	0.975
	**C.S**	66 (51.2%)	128 (51.0%)	194 (51.1%)	
**Vacuum**	**Yes**	18 (28.6%)	8 (6.5%)	26 (14%)	< 0.001
***N*** **= 186**	**No**	45 (71.4%)	115 (93.5%)	160 (86%)	
**Episiotomy**	**Yes**	38 (60.3%)	73 (59.3%)	111 (59.7%)	0.899
***N*** **= 186**	**No**	25 (39.7%)	50 (40.7%)	75 (40.3%)	
**Planned Pregnancy**	**Yes**	103 (79.8%)	181 (72.1%)	284 (74.7%)	0.1
	**No**	26 (20.2%)	70 (27.9%)	96 (25.3%)	
**Presence of Chronic disease**	**Yes**	12 (9.3%)	37 (14.7%)	49 (12.9%)	0.134
	**No**	117 (90.7%)	214 (85.3%)	331 (87.1%)	
**Rating of medical service**	**Excellent**	67 (51.9%)	101 (40.2%)	168 (44.2%)	0.123
	**Very well**	34 (26.4%)	75 (29.9%)	109 (28.7%)	
	**Good**	20 (15.5%)	41 (16.3%)	61 (16.1%)	
	**Acceptable**	5 (3.9%)	23 (9.1%)	28 (7.4%)	
	**Bad**	3 (2.3%)	11 (4.4%)	14 (3.7%)	

^+^ Chi-squared test; Significant level at *p* < 0.05

**Table 3:** Associations between Baby-related Factors and PPD

Variable	Categories	EPDS ≥ 13	EPDS < 13	Total	^**+**^***P*** value
**Sex**	**Male**	63 (48.8%)	137 (54.6%)	200 (52.6%)	0.288
	**Female**	66 (51.25)	114 (45.4%)	180 (47.4%)	
**Desired Sex**	**Male**	36 (18.3%)	59 (32.2%)	95 (25%)	0.531
	**Female**	36 (18.3%)	67 (36.6%)	103 (27.1%)	
	**No difference**	125 (63.4%)	57 (31.2%)	182 (47.9%)	
**Premature**	**Yes**	12 (9.3%)	24 (9.6%)	36 (9.5%)	0.935
	**No**	117 (90.7%)	227 (90.4%)	344 (90.5%)	
**NICU admission**	**Yes**	15 (11.6%)	22 (8.8%)	37 (9.7%)	0.373
	**No**	114 (88.4%)	229 (91.25)	343 (90.3%)	
**Illness**	**Yes**	2 (1.5%)	3 (1.2%)	5 (1.3%)	0.552^*^
	**No**	127 (98.5%)	248 (98.8%)	375 (98.7%)	
**Weight(kg)**	**≤ 2.5**	17 (13.2%)	36 (14.4%)	53 (13.9%)	0.853
	**2.51-4.49**	111 (86.1%)	214 (85.2%)	325 (85.5%)	
	**≥4.5**	1 (0.7%)	1 (0.4%)	2 (0.5%)	
	**Mdian:3.1, Range:3.6**				
**Feeding type**	**Breastfeeding**	47 (36.5%)	108 (43.0%)	155 (40.8%)	0.052
	**Formula**	20 (15.5%)	54 (21.5%)	74 (19.5%)	
	**Mixed**	62 (48.0%)	89 (35.5%)	151 (39.7%)	

^+^ Chi-squared test; Significant level at *p* < 0.05. *Fisher’s exact test

Most pregnancies occurred without complications (63%, *n* = 239) and in private hospitals (61.3%, *n* = 233) by Caesarean section (51.1%, *n* = 194). The majority of vaginal deliveries were not assisted by vacuum (86%, *n* = 160). However, of those that had a vaginal delivery, more than half underwent episiotomy repair (59.7%, *n* = 111).

Of the 380 women, 200 had boys (52.6%), 344 were full-term (90.5%), 375 were healthy (98.7%), with weights within the normal range (85.5%, *n* = 325). Most reported only breast feeding (40.8%, *n* = 155) or mixed with formula feeding (39.7%, *n* = 151).

### Psycho-social factors

Most participants reported no personal or family history of mental illness (98.4 and 77.4% respectively). Nearly half (47.9%) reported stressful life events during pregnancy factors such as financial stress and having additional children without family support are considered as risk factors for postpartum depression. The MSSS median was 20 (with a range of 22). Most women rated their families’ support in the medium range (53.7%, *n* = 204). See (Tables 4 and 5).

**Table 4:** Association between Psychological characteristics and PPD

Variable	Categories	EPDS ≥ 13	EPDS < 13 Depressed	Total	^**+**^***P*** value
**Personal history of mental illness**	**Yes**	1 (0.7%)	5 (2.0%)	6 (1.6%)	0.368
	**No**	128 (99.3%)	246 (98.0%)	374 (98.4%)	
**Treatment (*N*** **= 6)**	**Yes**	0 (0.0%)	2 (66.7%)	2 (33.3%)	0.667^*^
	**No**	3 (100.0%)	1 (33.3%)	4 (66.7%)	
**Family history of mental illness**	**Yes**	4 (3.1%)	14 (5.6%)	18 (4.7%)	0.555
	**No**	102 (79.0%)	192 (76.5%)	294 (77.4%)	
	**Don’t know**	23 (17.9%)	45 (17.9%)	68 (17.9%)	
**Stressful events in pregnancy**	**Yes**	78 (60.5%)	104 (41.4%)	182 (47.9%)	< 0.001
	**No**	51 (39.5%)	147 (58.6%)	198 (52.1%)	

^+^ Chi-squared test; Significant level at *p* < 0.05. *Fisher’s exact test

**Table 5:** Association between Social Support and PPD

Variable	Categories	EPDS > =13	EPDS < 13	N (%)	^**+**^*P* value
**MSSS**	**Low**	77 (59.8)	91 (36.2%)	168 (44.2%)	< 0.001
	**Medium**	51 (39.5%)	153 (61.0%)	204 (53.7%)	
	**Adequate**	1 (0.7%)	7 (2.8%)	8 (2.1%)	
	**Median: 20, Range: 22**				

^+^ Chi-squared test; Significant level at *p* < 0.05. MSSS: Maternal Social Support Scale

### Postpartum depression prevalence

At the time of our study 33.9% (*n* = 129) showed the risk of post-partum depression using the EDPS. The highest score was 28 and the lowest score was zero, with all answers negative for suicide attempts (Question 10 in the EPDS). The median score was 11.5 with a range of 28.

### Factors associated with PPD

Bivariate analysis (Chi-squared test and Fishers’ exact test as appropriate) was applied to all variables in the descriptive part of the results. Factors significantly associated with postpartum depression were lower level education of the husband, primiparity, vacuum use, stressful events during pregnancy, and low social support for the mother. A multi-logistic regression showed all factors remained significantly associated with PPD except primiparity. (See Table 6).

**Table 6:** Predictors of PPD by multivariate logistic regression

Factor		EPDS				Total	ORb 95% CI	*P*-Value
		≥ 13		< 13		N		
		N	%	N	%			
**Prim parity**	**Yes**	53	44.5%	66	55.5%	119	1.67 [0.99-2.8]	0.054
	**No(ref)**	76	29.1%	185	70.9%	261	1	
**Stressful events in pregnancy**	**Yes**	78	42.9%	104	57.1%	182	2.1 [1.27-3.4]	0.003
	**No(ref)**	51	25.8%	147	74.2%	198	1	
**Education (husband) (yrs)**	**≤ 6**	39	56.5%	30	43.5%	69	5.2 [2.7-10]	< 0.001
	**7-12**	45	34.6%	85	65.4%	130	1.7 [1.002-2.92]	0.049
	**> 12 (ref)**	45	24.9%	136	75.1%	181	1	
**MSSS**	**Low**	77	45.8%	91	54.2%	168	2.5 [1.7-4.2]	< 0.001
	**Medium (+) Adequate(ref)**	52	24.5%	160	75.5%	212	1	
**Vacuum use**	**Yes**	18	69.2%	8	30.8%	26	4 [1.64-9.91]	0.002
	**No(ref)**	45	28.1%	115	71.9%	160	1	

*OR* Odds Ratio; *CI* Confidence Interval, *ref*. reference level. ^**+**^Significant level at *p* < 0.05

Women experiencing stressful events during pregnancy were shown to be 2.1 times more likely to develop PPD (*p* value 0.003, OR: 2.1, 95% CI [1.27-3.4]), as did those with vacuum extraction of the baby (*p* value 0.002, OR: 4.0, 95%CI: [1.64-9.91]). Additionally, marriage to a man with a low level of education (6 years and less) increased the PPD five times more than marriage to a man with a high degree of education (more than 12 years) (*p* value less than 0.001, OR: 5.2, 95%CI: [2.7-10]).

Moreover, it was demonstrated that mothers with low social support showed a higher likelihood of experiencing PPD in comparison to mothers with both medium and adequate support (*p* value less than 0.001, OR: 2.5, 95%CI: [1.7-4.2]). Social support in both medium and adequate categories was calculated as one group due to low sample size in the adequate group.

## Discussion

Our study showed that prevalence of postpartum depression (women who scored 13 and more in the EPDS) was much higher than studies that used the EPDS in Nablus and Bethlehem which reported 17% an overall prevalence of PPD (8.9% scored 13 and more, 8.1% scored 10-12 on EPDS), 27.7% (score of 11 and more on the EPDS) respectively [8, 9]. The rise is not surprising and may be explained by the continued pressures Palestinians experience due to the restrictions and uncertainties imposed by being an occupied nation. Since the elected Palestinian government was politically and economically boycotted, living circumstances have become worse, leading to increased levels of unemployment, poverty, and internal conflict in Palestine as well as more limitations on access to healthcare [19] 18). These stressors result in mental instability and increase susceptibility to mental illnesses including PPD [1].

The increased prevalence of PPD in Palestine is similar to nearby Arab countries [20]. Surprisingly, Palestine’s prevalence of 33.9% is reasonable in comparison to what is published in nearby Arab region; recent systematic review study was held in the Arab region, 2020 and showed 8-40% range of PPD in Arab countries [10]. Another meta-analysis across the Middle East countries reported 27% prevalence [21]. In Saudi Arabia prevalence of PPD increased from 25.7% in 2017 to 38.5% in 2020 [22] 3). Another study in Damascus, 2017 reported a prevalence of 28.2% (EDPS score 13 and more) [23]. Higher prevalence was seen in Jordan; an article was published in 2021 and reported 52.9% prevalence between Jordanian women (EDPS score 12 and more) [24]. The global rise of PPD prevalence could be explained by the continuing COVID pandemic impact on health sectors both physically and mentally. Recent studies on the effect of the COVID-19 pandemic on PPD revealed increased levels of post-partum depression caused by increased fear [25, 26].

While reviewing the literature, we found variations in risk factors for PPD. Our study adds lack of social support, the husband’s low level of education, the occurrence of stressful events while pregnant and the use of vacuum as significant risk factors related to PPD. Our first two risk factors were also found in the Middle-East systematic review and meta-analysis [21]. However, stressful events in pregnancy or vacuum are new findings.

Vacuum extraction is reported by mothers as a negative experience which indirectly increases the possibility of experiencing PPD due to trauma to birth canal, post-delivery complications, increased pain and delay of return to normal activities [27]. All of this explains the significant association between vacuum and PPD which was reported in our study.

Another negative experience that showed significant association with PPD was stressful events during pregnancy, these includes mother argued with partner more than usual, separation, divorce, mother was in a physical fight, moved to new address, had a lot of unpaid bills, job loss, and close family member sick or died [28]. Several studies showed that stress increases amygdala activity which leads to mood changes and increasing probability of depression [29].

This study showed a significant association between low maternal social support and PPD; a new baby comes with many more duties and requirements, but assistance and support help mothers to cope faster [30]. Significant association between social support and PPD was also found in studies in nearby Arab countries with the same cultural and religious characteristics of Palestine [31].

Our significant negative association between higher levels of the husband’s education and PPD was also found in other studies across the world, India and East Turkey as an example [32, 33]. This can be contributed to the increased knowledge of women’s needs and the ability to provide good strategies of support. Here in Palestine, in order to provide acceptable living conditions, husbands with low educational level spend long working hours away from their wives limiting their ability to provide the needed support.

### Limitations

As a cross-sectional study, our findings don’t show causal relationships between variables. Although it is crucial and significant to have knowledge about the prevalence of postpartum depression in refugee and rural areas, there has been a lack of data collection in those areas. This can be attributed to budgetary constraints and the limitations imposed by the COVID-19 Act on UNRWA clinics in the period of data collection.

This resulted in a selection bias in participant selection, as the prevalence of PPD may increase in particular in refugee areas and still unknown in rural area. The study was conducted during the pandemic, a very difficult time across the globe and PPD may be higher due to the stresses of COVID. However, this study examines PPD prevalence and associated risk factors across a larger region in Palestine than prior studies.

## Conclusion

In conclusion, given the high prevalence of PPD in the northern of West Bank, Palestine, we recommend that antenatal clinics to expand their services from only maternal physical care to include mental health as well. Mothers should be screened for a range of stressors during pregnancy and efforts should be made to address them. This might include referrals to treat PPD with medication or offer therapeutic counseling support. Moreover, easy access to post-partum clinics should be offered for providing scheduled meetings including close family members to assure adequate social support especially for women whom vacuum was used during delivery or with stressful events during pregnancy.

### Recommendations

Create campaigns to educate the public about postpartum depression based on the findings of this study. At the national and regional levels, where mental health care services for women are limited, this should be emphasized. Policymakers and external funding agencies require this information to design future agendas.

## Data Availability

The datasets used and/or analysed during the current study available from the corresponding author on reasonable request.

## Abbreviations

PPD: Post-Partum Depression
EPDS: Edinburgh Postnatal Depression Scale
WHO: World Health Organization
MDE: Major Depressive Episode
SD: Standard Deviation
BDI: Beck Depression Inventory
MINI: Mini International Neuropsychiatric Interview
USPSTF: United States Preventive Services Task Force
OR: Odds Ratio
CI: Confidence Interval
